# Access Governance for Biobanks: The Case of the BioSHaRE-EU Cohorts

**DOI:** 10.1089/bio.2015.0124

**Published:** 2016-06-01

**Authors:** Jane Kaye, Linda Briceño Moraia, Colin Mitchell, Jessica Bell, Jasper Adriaan Bovenberg, Anne-Marie Tassé, Bartha Maria Knoppers

**Affiliations:** ^1^ HeLEX Centre for Health, Law and Emerging Technologies, Nuffield Department of Population Health, University of Oxford, Oxford, United Kingdom.; ^2^ Legal Pathways Institute for Health and Bio-Law, Aerdenhout, the Netherlands.; ^3^ Public Population Project in Genomics and Society (P3G), Montreal, Canada.; ^4^ Centre of Genomics and Policy, McGill University, Montreal, Canada.

## Abstract

Currently, researchers have to apply separately to individual biobanks if they want to carry out studies that use samples and data from multiple biobanks. This article analyzes the access governance arrangements of the original five biobank members of the Biobank Standardisation and Harmonisation for Research Excellence in the European Union (BioSHaRE-EU) project in Finland, Germany, the Netherlands, Norway, and the United Kingdom to identify similarities and differences in policies and procedures, and consider the potential for internal policy “harmonization.” Our analysis found differences in the range of researchers and organizations eligible to access biobanks; application processes; requirements for Research Ethics Committee approval; and terms of Material Transfer Agreements relating to ownership and commercialization. However, the main elements of access are the same across biobanks; access will be granted to bona fide researchers conducting research in the public interest, and all biobanks will consider the scientific merit of the proposed use and it's compatibility with the biobank's objectives. These findings suggest potential areas for harmonization across biobanks. This could be achieved through a single centralized application to a number of biobanks or a system of mutual recognition that places a presumption in favor of access to one biobank if already approved by another member of the same consortium. Biobanking and Biomolecular Resources Research Infrastructure-European Research Infrastructure Consortia (BBMRI-ERIC), a European consortium of biobanks and bioresources with its own ethical, legal, and social implications (ELSI) common service, could provide a platform by developing guidelines for harmonized internal processes.

## Introduction

Biobanks are repositories of tissue samples and clinical data, which are used for a range of research purposes and can be grouped into the broad categories of population-based prospective biobanks and disease-oriented biobanks.^1,2^ Accessing biobanks based in different jurisdictions maximizes the level of samples and data available for research with potential benefits for public health and also individual clinical care. Aspects of access raise ethical and legal questions as well as practical challenges.

There are general legal frameworks for data protection, which establish a set of common legal rules and principles for access and data sharing, as well as providing a number of exemptions for research. Within the research field, there have been a number of policy documents for sharing data (so-called external governance), which provide guidance that is specific for research and biobanking in particular.^3^ Drawing on these, biobanks have developed specific internal procedures and policies to govern access to their samples and data. In practice, it is these internal processes, such as contractual agreements, access procedures, and specialist committees, which play a pivotal role in the governance of access.^4^

The variation in access governance arrangements between biobanks has the potential to impede large-scale health research. This is because these arrangements are not designed from the perspective of the researcher, who must invest significant time and effort applying to each biobank separately without being certain of the nature of samples or data available for research until all the biobanks respond. However, it may be possible to develop a level of “harmonization” of internal governance arrangements, which can respect the sovereignty of each biobank within the national and international legal framework, rather than attempting to develop biobank-specific national laws or guidance.

The need for such integration is emphasized by the European Commission as “essential to obtain the large numbers of participants and samples necessary to conduct research investigating, for example, the interplay between genetic, lifestyle, environmental and social factors that determine health and (complex) diseases” (6, ch. 4). Although, as we demonstrate below, there are differences between biobanks in terms of their objectives and requirements, this form of research is within the scope of all biobanks studied here.

This article analyzes the access governance arrangements of the five original biobank members of the Biobank Standardisation and Harmonisation for Research Excellence in the European Union (BioSHaRE-EU) project^5^ to identify the main similarities and differences in policy and procedure, and makes recommendations for increased harmonization of internal governance among European biobanks. BioSHaRE-EU is a pan-European research consortium that aims to facilitate data sharing across multiple biobanks and databases, with a focus on the scientific, bioinformatics, and ethical and legal elements of harmonization and standardization. Its biobank members are all legally independent entities spread across eight European countries. They provide a case study for the main similarities and differences in governance of access in well-developed cohorts and biobanks.

The law in this area is currently nationally fragmented. Even the newly adopted EU General Data Protection Regulation, which harmonizes some aspects of data protection, will allow Member State derogations—permitted variations—on specific issues such as processing of health data for research purposes without consent, suggesting that further national variation of data protection law is likely. Such fragmentation contrasts with soft-law and guidance that advocates harmonization of access to biobanks.^6^ This article argues that it may be possible to streamline access policies and procedures within the pan-European research infrastructure, Biobanking and Biomolecular Resources Research Infrastructure-European Research Infrastructure Consortia (BBMRI-ERIC), proposing a form of “harmonized” internal governance of biobanks.

### The policy framework for biobanking

In recent decades, international bodies have sought to identify and express fundamental principles of access in health research in general policies such as the OECD Principles and Guidelines for Access to Research Data from Public Funding,^7^ the Bermuda Principles in 2003,^8^ Fort Lauderdale Agreement in 2003,^9^ as well as the Toronto Statement in 2009,^10^ which developed a set of suggested “best practices” for funding agencies, scientists, and journal editors. More recently, the Framework for Responsible Sharing of Genomics and Health-Related Data (2014) of the Global Alliance for Genomics and Health^11^ reassessed these principles with a human rights perspective; and the Nuffield Council on Bioethics released a report on the ethical issues around the collection, linking, and use of data in healthcare and biomedical research.^12^ In the United Kingdom, the Expert Advisory Group on Access (EAGDA) established by the Medical Research Council, the Economic and Social Research Council, Cancer Research UK, and the Wellcome Trust has also published a report on “Governance of Access,” to enhance the dissemination of good practice across the funders.^13^

A number of specific guidelines of best practices for access in biobanking and genetic research databases have also been developed, such as the OECD Guidelines on Human Biobanks and Genetic Research 2009^14^ and the HUGO statement on Human Genetic Databases 2002.^15^ These policies recommend that biobanks should have as a minimum a transparent access policy that is readily available for participants, researchers, and third parties using the biobank. Recommendations for the contents of access agreements include the following: the nature of the material available; the purposes for which the material may be used; requirements for additional ethical review; intellectual property arrangements; access fees; ownership or “custodianship” of the material; consent provisions; requirements to ensure confidentiality; and limits on users and uses of materials.

Recognizing the varying regulatory approaches of different jurisdictions toward access in biobanks, internal governance procedures such as material transfer agreements (MTA) have thus far been prioritized by the ethical community and research consortia to authorize the transfer of human biological materials and data outside the geographical jurisdiction from which they originated. Within the framework of international research consortia projects, researchers have developed a *Charter of principles for sharing bio-specimens and data*, together with a template for general MTA.^16^ These documents aim to improve the governance and audit of sharing data and specimens across different jurisdictions. There have also been proposals for a harmonized access agreement, such as the P3G Generic Access Agreement,^17^ which addresses both the sharing of data and samples and offers a template to assist or inform researchers seeking access to population studies.

### The BBMRI–ERIC for European Biobanking

ERICs were established by the European Union in 2009 as a means of promoting and furthering research in Europe.^18^ They are funded by the European Union, Member States, and other associated states but exist as separate international organizations, although with lines of accountability and reporting to the European Commission. On this basis, the European Commission established the Biobanking and Biomolecular Resources Research Infrastructure as an ERIC (BBMRI-ERIC) to integrate quality controlled biobanks and bimolecular resources across Europe, in 2013.

The field of research and health is one of limited competence for the European Union; it shares competence with Member States in the field of research (this means both may legislate on these issues according to Art. 4 TFEU) and may only harmonize law in this area, subject to the principles of subsidiarity and proportionality, where the goal of regulation cannot be better achieved at national levels. For this reason, BBMRI-ERIC is not a platform for legal harmonization but may provide a Europe-wide opportunity for the development of streamlined approaches and soft-law governing research and access to data, which Member States could accommodate or adopt within their own legal frameworks.^19^

This article analyzes the access governance arrangements of the original five biobank members of the BioSHaRE-EU project to identify similarities and differences in policies and procedures, and consider the potential “harmonization” of aspects of data governance through structures such as BBMRI-ERIC.

## Methods

The BioSHaRE-EU project involved fourteen biobanks. In this article, we analyze the formal data access governance arrangements of the five original BioSHaRE—EU biobanks—Finrisk,^20^ KORA,^21^ LifeLines,^22^ HUNT,^23^ and UK Biobank.^24^ As well-established biobanks with well-developed governance arrangements, these provide a case study of similarities and differences within the EU legal framework. The key similarities and differences are identified below. Information on access requirements for each biobank was provided by colleagues within the jurisdictions of each biobank, as well as identified through publically available policy documents and regulations.

## Results

The five original BioSHaRE-EU biobanks are very similar in their governance structures and funding as they are all prospective biobanks or longitudinal studies. The FINRISK Study is a population survey on risk factors on chronic, noncommunicable diseases and it is based on a project that was part of the World Health Organization Monica Project. The UK Biobank is long-term population study investigating the impact of genetic predisposition and environmental exposure, and it is a registered charitable company funded by the Department of Health and other organizations. KORA is also financed by public funds allocated by the Federal Ministry of Education and Research and the State of Bavaria, and it is focused on the study of the development and course of chronic diseases.

HUNT includes different population surveys carried out in different periods and it is funded by the Norwegian Institute of Public Health. LifeLines is funded by the University Medical Center Groningen and it has a broad scope, encompassing studies on epigenetic, biomedical, environmental, and psychosocial factors in relation to healthy aging, disease development, and general well-being.

The BioSHaRE-EU biobanks share a similar organizational nature, as in all cases, they are based, totally or partially, on public funding, which is typical of many biobanks. The nature of the material each biobank makes available is similar in the BioSHaRE-EU biobanks. For example, FINRISK studies are based on cross-sectional population surveys and there are different types of biosamples available such as DNA, serum, and plasma. KORA-gen is a platform to provide phenotypes, genotypes, and biosamples for collaborative genetic epidemiological research; LifeLines comprises a longitudinal cohort and a biobank, including a data warehouse and biomaterial.

### The access requirements of the BioSHaRE-EU biobanks

As BioSHaRE-EU biobanks are all legally independent entities, researchers need to contact each cohort individually to obtain access authorization. Access policies differ across these biobanks. In some cases, biobanks do not set out information in a specific access policy, but the essential requirements are found in other documents. For example, access information in KORA can be found in the project's agreements for data transfer, while informative brochures are freely available on the HUNT biobank website, where participants may also find all the relevant information regarding the whole process (consent and project approval, sample collection and storage, and ethics approval). For participation in Lifeline, the adult protocol describes the program for adults participating in the study (information about the study, methods, consent, and ethical considerations). The UK Biobank has a comprehensive Access Policy, governed by principles enshrined in the Biobank's self-regulatory Ethics and Governance Framework and monitored by the specifically created UK Biobank Ethics and Governance Council.

#### Who has access?

The spectrum of the persons entitled to access the data may vary depending on the biobank and this is reflected in the BioSHaRE-EU biobanks. Data sharing policy guidance recommends that biobanks must indicate to donors the uses allowed and the purposes under which researches can be conducted so that participants may provide an informed consent. In some cases, access may be limited to nonprofit institutions (such as universities, charities, public bodies), while others allow access to commercial companies. For example, the UK Biobank allows commercial companies to access data, as long as it is for conducting research in the “public interest.” Researchers from the private sector are not eligible to access data of the FINRISK biobank, while LifeLines welcomes other public and private partners. Currently, researchers have to apply individually for access rather than institutional agreements being in place. This gives greater control to the biobank managers as to who can access samples and data rather than this being the responsibility of the researcher's institution. This places the responsibility for oversight largely on the biobank.

#### Who is responsible for access?

Most biobanks have a body appointed to review access applications, usually known as data access committees (DACs). The composition and the nature of the DACs may vary across the different biobanks with no set formula; often the public or participants are not represented, but there may be nonscientific representation. For example, UK Biobank's data access subcommittee is currently chaired by a lawyer (www.ukbiobank.ac.uk/access-to-the-resource). LifeLines' Scientific Board consists of principal investigators who have the responsibility, among others, to secure the scientific quality and utility of the collected data and to review submitted research proposals for use of LifeLines data. The review of the access requests for KORA biobank is performed by a Steering Committee, which will approve applications within 3 weeks. Within the UK Biobank, the Board of Directors has overall responsibility for the access procedures and all access decisions. It delegates oversight of the review process to its Access Subcommittee. In the case of the FINRISK Study, the research plan must be approved by a committee at the National Institute for Health and Welfare, and linkages to other registries require separate applications for permission. There are significant differences between BioSHaRE-EU biobanks as the DAC may be internal or external to the biobank,^25^ and the list of the members may or may not be publicly available. Sometimes biobanks have a scientific committee with the overall decision-making authority over access and use of the resource and an advisory board, an independent body that advises the scientific committee on governance issues (www.generationscotland.org/images/stories/GS_MAPP.pdf).

#### What is the access procedure?

**FIG. 1.**
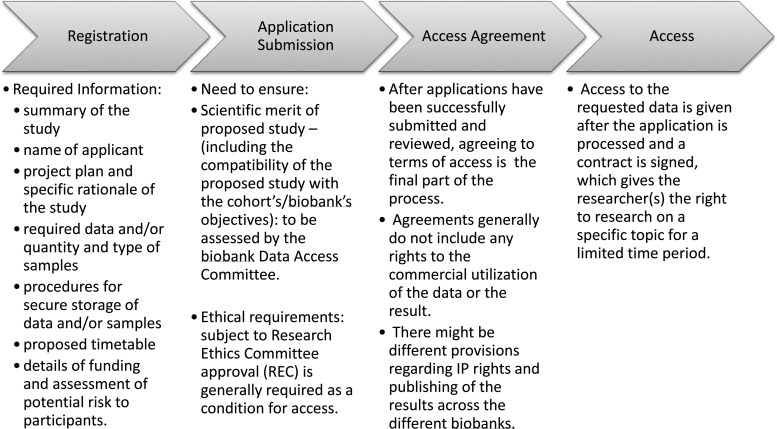
Summary of access procedures.

Three steps can be identified in the application process for access across these biobanks; registration, application, and agreement to terms of access (Fig. 1).

#### Registration

Registration aims to verify the identity of researchers who wish to have access to a biobank and to confirm their bona fides before being recognized as an approved researcher. This is generally a common requirement, but the procedures for registering may differ from one biobank to another. For example, in the Norwegian HUNT biobank, researchers do not register directly with the biobank (www.ntnu.edu/hunt/data) but with the Norwegian Social Science Data Services (NSD). This is a separate public body linked to the Ministry of Education and Research providing assistance with data gathering, data analysis, and issues of methodology, privacy, and research ethics.^26^ Another model is that of UK Biobank, where researchers register and have a relationship with UK Biobank through an online portal (https://amsportal.ukbiobank.ac.uk/Pages/Registration.aspx) and guidance is provided by the biobank on their website (www.ukbiobank.ac.uk/register-apply). As an example of the criteria used to assess whether an applicant is a bona fide researcher, the UK Biobank registration form requires a CV, list of peer-reviewed publications, and research department contact information so that the biobank can confirm identity and position of the applicant.^27^

#### Application

Next is the application phase where the scientific merit of the proposed study will be scrutinized, addressing such issues as the compatibility of the proposed study with the cohort/biobank's objectives and whether there has been approval for the research by a Research Ethics Committee (REC). The way in which an application is evaluated varies across biobanks. Most biobanks have a single-stage application, with the exception of UK Biobank, which requires a “preliminary application” to help researchers determine their likelihood of success and indicative costs. As part of an application, the majority of BioSHaRE-EU biobanks require separate REC approval. However, there is no European law requiring this, and there is some variation among the biobanks.^6^ Notably, UK Biobank has to obtain Research Tissue Bank (RTB) approval from its governing REC, which means that, for the “great majority” of proposed uses of the resource, researchers will not need to obtain separate ethics approval.^27^ The application process frequently involves communication between the researcher and the biobank with an opportunity to provide further clarification and information to assist the access committee's decision. If an applicant is advised that the biobank is inclined to decline access, there may be an opportunity to request that an application is reconsidered. For example, UK Biobank allows a request for reconsideration with reasons within 3 months of a decision.

#### Access agreement

After the access application has been successfully submitted, reviewed, and approved, the access agreement is the final step of the request process. The contractual relationship between partners may differ. In some cases, the contracting party is the single researcher (e.g., in UK Biobank), and in other cases, it is the researcher's institution that appoints a principal investigator for each project (as with the Norwegian HUNT Biobank). An agreement will also generally detail the purposes for which the biobank may be accessed, including the commercial utilization of the data or the result. For example, although FINRISK biobank does not allow access to researchers from the private sector, it encourages cooperation under an intellectual property perspective since it recognizes coauthorship, patent sharing, as well as the return of data and results to the individual biobank/study. A similar collaborative approach is also reached through licensing agreements; for example, the UK Biobank is the unique owner of the intellectual property rights in the data in the resource, and it grants the applicant a limited, revocable, worldwide, royalty-free, nonexclusive license (but not any ownership rights) to use the samples and data for the permitted purpose only. The Dutch LifeLines biobank provides an exclusive right to use the data for a predetermined period of time and scientific results become publically available.

## Discussion

Within the BioSHaRE-EU biobanks, there are a number of different access requirements, which means it is currently not possible for researchers to apply through a single system to access samples and data from a number of biobanks based in different countries. The biobanks in the BioSHaRE-EU project have developed their access policies independently over a number of years, in different countries across Europe, according to national laws, procedures, and norms. This has resulted in differences in the composition and the nature of DACs, which researchers need to be aware of. These differences have implications for researchers.

First, the type of researchers and the range of organizations eligible to access the biobank resources vary; in some cases, commercial companies may access data if they are conducting research in the “public interest” (UK Biobank), whereas researchers from the private sector are not eligible to access the FINRISK biobank.

There are also differences in access procedures that researchers must follow. In some cases, preapplication advice is provided by a purpose-built body, for example, LifeLines biobank's expert center, whereas in UK Biobank, a “preliminary” application stage provides an early check for researcher's suitability, likelihood of success, and anticipated costs. Most BioSHaRE-EU biobanks require a single main application and within this application, researchers may have to fulfill different criteria.

REC approval is required by most biobanks and cohorts within BioSHaRE-EU. The variation in approaches of local RECs may form a significant barrier to access to multiple biobanks. This is not a barrier in all countries, however; within the UK legal and ethical framework, the great majority of proposed uses of data held by UK Biobank are covered by its own “generic” REC approval as long as the terms of this are reflected in the MTA. This may well streamline the process for researchers, but could have implications in the future if access procedures are harmonized across biobanks according to a minimum standard that reflects the majority.

The terms of MTAs themselves also vary between biobanks, with particular differences relating to ownership and commercialization. Despite these differences, on the basis of our analysis, there are some possibilities for improving the system of access governance across European biobanks. As data access requirements move away from mere requests to principal investigators and more formalized governance structures are put in place, our analysis identifies similarities and differences to biobanks that will aid the international biobank community in developing harmonized approaches to governance of access.

The main elements of access are the same across the BioSHaRE-EU biobanks; bona fide nonprivate sector researchers conducting research in the public interest are entitled to access and must register in some way to do so. The application process in all cases will consider the scientific merit of the proposed use and determine compatibility with the cohort or biobank's objectives. These are elements that could be harmonized across the biobanks. Perhaps more fundamentally, a central body providing preapplication guidance to researchers and perhaps even a single centralized application system at a supranational level could greatly improve the efficiency of access to multiple biobanks.

The BioSHaRE-EU project has introduced some harmonization of data access to streamline the process of applying to multiple cohorts. A researcher is able to apply to a central Access Coordination Committee (ACC) and their harmonization team who ensure the feasibility of harmonizing variables and performing analysis using DataSHIELD. A central IT team can help to develop processing algorithms that local IT teams install and use. This procedure focuses solely on IT harmonization and excludes all ethical and legal considerations, which are still considered by local DACs individually. This centralized support can increase efficiency of applying to multiple biobanks, but within the current regulatory framework, researchers still need approval from each cohort.

However, new technologies may also unlock the potential to develop e-governance^28^ systems, which could facilitate access in compliance with legal requirements across territorial boundaries.^29^ They would enable connection between the wider community of researchers and the biobanks and vice-versa, without necessarily substituting the specific national access processes. Instead, they could represent a different solution for cross border research. Biobanks could be propelled to adhere to such a system as a way to create new forms of collaborations and potentially find new opportunities to address the sustainability challenge. This would enable researchers to access multiple data sets using a “fast-track” procedure, which could be designed following common standards and in compliance with the relevant data protection rules. Such a system could be implemented within existing international platforms.

While streamlining governance of access may not be a straightforward task, there are already a number of platforms that present opportunities for improvement as well as incentivizing individual biobanks to join these efforts. An important potential platform for increased harmonization, centralization, and e-governance is BBMRI-ERIC.^30^ This European consortium of biobanks and bioresources is governed by an assembly of members and their appointed director-general.^31^ Within its own statutes, BBMRI-ERIC has established an ELSI Common Service,^32^ which monitors ELSI issues related to biobanks and could provide proposals for addressing joint matters for the biobanking community on the European level (http://bbmri-eric.eu/common-services). BBMRI-ERIC is also obliged to report annually to the European Commission, including any circumstances that threaten to jeopardize the achievement of the task of the ERIC (Art. 17 of the ERIC regulation). While BBMRI-ERIC could not develop legal proposals for harmonization of access, it could influence the EU's proposals in this area and perhaps,^19^ through the ELSI common service, develop some harmonized internal processes for access that could be adopted by constituent biobanks. BBMRI-ERIC incentivizes biobanks to become a member by offering a series of tools and services as well as expertise and support for biobanking. Such incentives may play an important role in encouraging individual biobanks to harmonize governance arrangements in the future.

Although a partially centralized and harmonized system of access might make health research across Europe more efficient, this may take time to develop. More immediate term solutions could be designed acknowledging diversity across biobanks by providing access to data across a group of biobanks, such as the BioSHaRE-EU biobanks, on the basis of a mutual recognition of application approvals between biobanks. Such a system might place a presumption in favor of access to data in one biobank when an application has already been granted by a partner resource. Local scrutiny might not be done away with entirely but a presumption in favor and a lighter review could be considered a form of “proportionate governance” of access. While the existing system has a number of benefits in terms of maintaining control of oversight by the individual biobank, there is the potential to streamline common access procedures across biobanks in Europe to facilitate research that requires samples and data from a number of biobanks. BBMRI-ERIC has the potential to greatly assist this process by developing some guidelines for harmonized internal processes.
